# Photon Optimizer (PO) prevails over Progressive Resolution Optimizer (PRO) for VMAT planning with or without knowledge‐based solution

**DOI:** 10.1002/acm2.12038

**Published:** 2017-01-24

**Authors:** Fan Jiang, Hao Wu, Haizhen Yue, Fei Jia, Yibao Zhang

**Affiliations:** ^1^ Key Laboratory of Carcinogenesis and Translational Research (Ministry of Education/Beijing) Department of Radiation Oncology Peking University Cancer Hospital & Institute Beijing Cancer Hospital Beijing China; ^2^ Department of Radiation Oncology The First Affiliated Hospital of Zhengzhou University and Department of Radiation Oncology Basic Medical College of Zhengzhou University Zhengzhou Henan China

**Keywords:** knowledge‐based planning, Photon Optimizer, Progressive Resolution Optimizer, RapidPlan, VMAT

## Abstract

The enhanced dosimetric performance of knowledge‐based volumetric modulated arc therapy (VMAT) planning might be jointly contributed by the patient‐specific optimization objectives, as estimated by the RapidPlan model, and by the potentially improved Photon Optimizer (PO) algorithm than the previous Progressive Resolution Optimizer (PRO) engine. As PO is mandatory for RapidPlan estimation but optional for conventional manual planning, appreciating the two optimizers may provide practical guidelines for the algorithm selection because knowledge‐based planning may not replace the current method completely in a short run. Using a previously validated dose–volume histogram (DVH) estimation model which can produce clinically acceptable plans automatically for rectal cancer patients without interactive manual adjustment, this study reoptimized 30 historically approved plans (referred as clinical plans that were created manually with PRO) with RapidPlan solution (PO plans). Then the PRO algorithm was utilized to optimize the plans again using the same dose–volume constraints as PO plans, where the line objectives were converted as a series of point objectives automatically (PRO plans). On the basis of comparable target dose coverage, the combined applications of new objectives and PO algorithm have significantly reduced the organs‐at‐risk (OAR) exposure by 23.49–32.72% than the clinical plans. These discrepancies have been largely preserved after substituting PRO for PO, indicating the dosimetric improvements were mostly attributable to the refined objectives. Therefore, Eclipse users of earlier versions may instantly benefit from adopting the model‐generated objectives from other RapidPlan‐equipped centers, even with PRO algorithm. However, the additional contribution made by the PO relative to PRO accounted for 1.54–3.74%, suggesting PO should be selected with priority whenever available, with or without RapidPlan solution as a purchasable package. Significantly increased monitor units were associated with the model‐generated objectives but independent from the optimizers, indicating higher modulation in these plans. As a summary, PO prevails over PRO algorithm for VMAT planning with or without knowledge‐based technique.

## 1 Introduction

Using the anatomical structures, field geometry, dose metrics, and dose prescription of previous plans as historical experiences to predict the dose–volume objectives for the upcoming patients,^1^, ^2^ knowledge‐based treatment planning has been deemed as a promising solution to reduce the subjective inter‐planner varieties,^3^, ^4^, ^5^, ^6^, ^7^, ^8^, ^9^, ^10^ enhance the clinical efficiency and quality of prospective plans.^2^, ^11^, ^12^, ^13^, ^14^, ^15^ As a commercial knowledge‐based optimizer, RapidPlan in Eclipse treatment planning system (V13.5 or later, Varian Medical Systems, Palo Alto, CA, USA) has been validated by many publications suggesting superior dosimetric outcomes than the conventional methods.^16^, ^17^, ^18^, ^19^, ^20^, ^21^, ^22^, ^23^ However, in addition to the usage of possibly advanced personalized optimization objectives as generated by the dose–volume histogram (DVH) estimation models, the new Photon Optimizer (PO) was introduced to combine and substitute for the two old algorithms, that is, Progressive Resolution Optimizer (PRO) for volumetric modulated arc therapy (VMAT) and Dose Volume Optimizer (DVO) for static field intensity‐modulated radiotherapy (IMRT). By far, no study has been conducted to confirm if the observed dosimetric progress was also partially (if any) attributable to the potentially better PO algorithm.

According to the manufacturer, DVO optimizes the field shape and intensity using a simple gradient optimization to approach the desired dose–volume objectives. The fluences are back‐projected from the derivates of the costs at each cloud point representing the patient volume. PRO optimizes MLC leaf positions and MU/deg based on control points segmentation of the gantry angle. As the optimization progresses, the accuracy of the angle resolution and dose calculation segments increase. Relative to prior optimizers, PO has involved critical changes: the old point cloud model for PRO and DVO has been replaced by a new volume representation, where “structures, DVH calculation and dose sampling are defined spatially by using one single matrix over the image”. However, DVO and PRO have a more powerful dose–volume objective form than the PO in terms of limiting the local minima in the optimization space. More technical details were described by Vanetti et al.^24^ and Cozzi et al.^25^ It is noticed that PO is mandatory for RapidPlan to accommodate its geometry‐based expected dose (GED) algorithm, yet is optional for the conventional manual planning where all the optimization features can be used in the same way without a model.

Providing knowledge‐based planning is not likely to replace the conventional methods completely in a short run, appreciating the behavior of the new PO against the old optimizers may provide useful guidelines for algorithm selection when RapidPlan is not invoked as a separate executable option. After all, as a purchasable engine, RapidPlan is not available to every Eclipse user, and the model configuration can be a sorely time‐consuming process. In order to assess the different algorithms based on the same (but patient specific) optimization objectives that can produce clinically acceptable plans without interactive manual adjustment, all DVH constraints were derived from our RapidPlan model estimation that has been validated for rectal cancer patients.^16^, ^17^ However, this model was configured with features that are not supported by DVO, such as mean dose objective and automatic normal tissue objective; hence, this study only focuses on VMAT planning using PO and PRO algorithms, respectively.

## 2 Methods

This study was conducted based on Varian Eclipse Treatment Planning System V. 13.5.

### 2.A DVH estimation model

As a brief summary of the previous work,^16^, ^17^ a DVH estimation model was configured using 81 historical VMAT plans for preoperative rectal cancer patients with simultaneous‐integrated‐boosting (SIB). The library size was determined based on Boutilier's study and the rule‐of‐thumb approach.^26^ The gross target volume (GTV) was delineated to cover the primary tumor, mesorectal space, and the involved lymph nodes.^27^ The clinical target volume (CTV) included GTV, presacral region, mesorectal/lateral lymph nodes, internal iliac lymph node chain, and pelvic wall area. Should the anterior organ involvement was suspected, CTV also includes the external iliac lymph nodes, and includes the inguinal lymph nodes when the lower third of the vagina was invaded or major tumor extension into the internal and external anal sphincter was observed.^28^ Isotropic margins of 5 mm were applied to create planning target volumes (PTV_boost_ from GTV, and PTV from CTV, respectively). Target dose of 50.6 Gy and 41.8 Gy were prescribed to 95% of PTV_boost_ and PTV in 22 fractions, respectively. All plans were created with 1–2 full arc, 5° collimator rotation, and 10 MV photon beams modulated by Varian Millennium 120 multi‐leaf collimator mounted on a Varian Trilogy accelerator. As reported before, the model validation on over 100 historical plans displayed significantly improved dosimetric results than the average clinical level, after applying the RapidPlan‐generated objectives and the PO algorithm.

### 2.B VMAT planning using PO and PRO

Thirty clinical VMAT rectal plans that have been manually optimized with PRO were retrospectively selected and reoptimized using the RapidPlan engine (referred as PO plans). Representing the average planning quality, these 30 consecutively treated plans were not used for model development but followed consistent contouring^28^ and planning protocols as for the training set. Although model estimations are only available with PO, the objectives and priorities calculated from the estimation are applicable to PRO, where the line objectives are represented as a series of points (referred as PRO plans). This approach ensured the clinically acceptable PO, and PRO plans were derived from identical constraints and objectives without interactive human adjustment during the optimization process. All other parameters were maintained as earlier during the optimization as well; hence, the observable discrepancies were mostly ascribed to the disparities of PO and PRO algorithms. Renormalization was performed to assess the organs‐at‐risk (OAR) sparing based on comparable target dose coverage (i.e., 95% PTV_boost_ and PTV were covered by corresponding prescribed dose, respectively).

### 2.C Dosimetric evaluation and statistical method

The clinical, PO and PRO plans were evaluated mutually by means of the target homogeneity index (HI_PTVboost_ and HI_PTV_); the target conformity index (CI_PTVboost_ and CI_PTV_); dose to 50% of the volume for the femoral head and urinary bladder (D_50%_FH_ and D_50_UB_), the mean dose (D_mean_FH_ and D_mean_UB_); the hot spot volume receiving over 107% of the prescribed dose to PTV_boost_ (V_107%_, i.e., V_54.14Gy_), and the total monitor units (MU). Based on SPSS 21 software (IBM Analytics, Armonk, NY, USA), paired samples *t*‐test was used to analyze the normally distributed data (Shapiro–Wilk method); otherwise Wilcoxon signed‐rank test was conducted. Significant level was put at *P* < 0.05 (2‐tailed).

## 3 Results

Without interactive human interference, the patient‐specific optimization objectives generated from the model estimation functioned well with both PO and PRO algorithms, which produced clinically acceptable plans automatically as visually inspected on three‐dimensional dose color wash and DVH distribution.

Numerical dosimetric comparison of plans using various techniques are listed in Tables 1 (target coverage) and 2 (OAR sparing). Relative to the median values, the mean dose deviated by 0.12 Gy and 0.17 Gy to PTV_boost_, and deviated by 0.34 Gy and 0.09 Gy to PTV, respectively, among different planning techniques. The inter‐group disparities of HI_PTVboost_, HI_PTV_, and CI_PTV_ were all within 0.01–0.02. Clinical plans achieved significantly lower CI_PTVboost_ (differed by 0.05–0.06), yet the values for PO and PRO groups were largely comparable (varied by 0.01 only, *P* > 0.05). Relative to the clinical plans, the usage of RapidPlan‐generated optimization objectives (PO and PRO cases) has also induced: (a) Higher MUs (by 7.92% and 8.17% for PO and PRO); (b) Significant reduction of D_50%_FH_, D_mean_FH_, D_50%_UB_, and D_mean_UB_; (c) Significant reduction or elimination of V_107%_.

**Table 1:** Dosimetric comparison (target coverage) of 30 patients that were planned by: PRO using the manual objectives as in the clinical plans (clinical); PO using the RapidPlan‐generated objectives (PO); and PRO using the RapidPlan‐generated objectives (PRO). Dose unit (Gy)

		Mean	SD	95% Confidence interval		*P*
				Lower	Upper	
D_PTVboost_	Clinical	52.22	0.19	52.15	52.29	<0.01^a^
	PO	51.93	0.23	51.85	52.01	0.03^a^
	PRO	52.05	0.30	51.94	52.16	
D_PTV_	Clinical	45.51	0.79	45.22	45.81	<0.01
	PO	45.85	0.75	45.57	46.13	0.19
	PRO	45.94	0.72	45.67	46.21	
HI_PTVboost_	Clinical	0.06	0.01	0.06	0.06	<0.01
	PO	0.05	0.01	0.05	0.05	0.53
	PRO	0.05	0.01	0.04	0.05	
HI_PTV_	Clinical	0.26	0.01	0.26	0.26	<0.01
	PO	0.25	0.01	0.25	0.26	<0.01
	PRO	0.26	0.01	0.26	0.26	
CI_PTVboost_	Clinical	1.01	0.03	1.00	1.02	<0.01^a^
	PO	1.06	0.05	1.05	1.08	0.60^a^
	PRO	1.07	0.05	1.05	1.09	
CI_PTV_	Clinical	1.04	0.03	1.03	1.05	0.34
	PO	1.05	0.03	1.04	1.06	<0.01^a^
	PRO	1.03	0.03	1.02	1.04	
MUs	Clinical	404	30	392	415	<0.01
	PO	436	33	423	448	0.81
	PRO	437	29	426	448	

a Wilcoxon signed‐rank test (abnormal distribution); otherwise paired samples t‐test (normal distribution).

D_PTVboost_, mean dose to PTV_boost_; D_PTV_, mean dose to PTV; HI, homogeneity index; CI, conformity index; SD, standard deviation; and MUs, monitor units.

**Table 2:** Dosimetric comparison (OAR sparing) of 30 patients that were planned by: PRO using the manual objectives as in the clinical plans (clinical); PO using the RapidPlan‐generated objectives (PO); and PRO using the RapidPlan‐generated objectives (PRO). Dose unit (Gy)

		Mean	SD	95% Confidence interval			*P*
				Lower		Upper	
D_50%_FH_	Clinical	15.10	2.28	14.25		15.95	<0.01
	PO	10.16	2.65	9.18		11.15	0.06
	PRO	10.43	2.95	9.33		11.53	
D_mean_FH_	Clinical	16.27	2.10	15.49		17.05	<0.01
	PO	12.31	1.71	11.67		12.95	<0.01
	PRO	12.56	1.87	11.86		13.25	
D_50%_UB_	Clinical	28.05	2.60	27.08		29.02	<0.01^a^
	PO	19.18	1.96	18.45		19.91	<0.01
	PRO	20.23	2.05	19.47		21.00	
D_mean_UB_	Clinical	29.21	2.10	28.42		29.99	<0.01
	PO	22.35	2.02	21.59		23.10	<0.01
	PRO	23.12	2.09	22.34		23.90	
V_107%_	Clinical	0.04	0.11	0.00		0.08	0.01^a^
	PO	Not observed					0.32^a^
	PRO	0.01	0.05	‐0.01		0.03	

Wilcoxon signed‐rank test (abnormal distribution); otherwise paired samples *t*‐test (normal distribution).

a SD, standard deviation; D_50%_, dose to the 50% volume of the structure; D_mean_, mean dose; FH, femoral head, UB, urinary bladder, and V_107%_, volume receiving over 107% of the prescribed dose.

**Figure 1:**
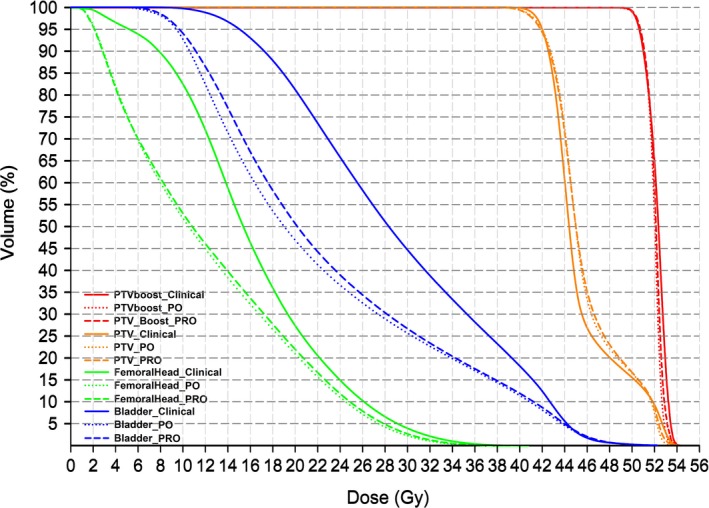
The mean DVHs of 30 patients: the solid, dotted, and dashed lines indicate the clinical, PO and PRO plans, respectively.

Figure 1 displays the mean DVH plots of the 30 patients as grouped by the plan types. The clinical, PI and PRO plans are plotted as solid, dotted, and dashed lines, respectively. Adequate target dose coverage (prescription: 50.6 Gy and 41.8 Gy to 95% of PTV_boost_ and PTV, respectively) was achieved by all three techniques, yet the dose to the OARs was significantly reduced using RapidPlan‐generated objectives (PO and PRO plans) than the manual settings (clinical plans).

Taking the clinical plans as baseline, Table 3 breaks down the relative improvement of OAR sparing as contributed by the new objectives and the optimizers, which differs by an order of magnitude.

**Table 3:** A breakdown of relative contributions to the improved OAR sparing by the new objectives and optimizers, where total improvement = (D_clinical_−D_PO_)/D_clinical_; objective contribution = (D_clinical_−D_PRO_)/D_clinical_; and optimizer contribution = (D_PRO_−D_PO_)/D_clinical_

	D_50%_FH_	D_mean_FH_	D_50%_UB_	D_mean_UB_
Total improvement (%)	32.72	24.34	31.62	23.49
Objective contribution (%)	30.93	22.80	27.88	20.85
Optimizer contribution (%)	1.79	1.54	3.74	2.64

D_50%_, dose to the 50% volume of the structure; D_mean_, mean dose; FH, femoral head, UB, urinary bladder; D_clinical_, D_PO_ and D_PRO_, dose parameters corresponding to the clinical, PO and PRO plans.

## 4 Discussion

With adequate dose coverage to 95% PTV_boost_ and PTV by renormalization, PO and PRO methods have significantly lowered the target mean dose than clinical plans, indicating reduced hot spots in the targets and improved dose homogeneity (Table 1). Although statistically significant, the magnitudes of the numerical difference were relatively small; hence, may be clinically negligible, as echoed by Fig. 1, demonstrating that prescription was selected, such that no difference between the clinical plans and reoptimized plans occurred to bias any results with the OAR dose comparison. The similar CI_PTVboost_ between the PO and PRO plans suggested that the smaller value of the clinical plans should be mostly attributable to different objective settings rather than the optimization algorithms.

Consistent with earlier publications,^10^, ^14^, ^15^, ^16^, ^17^, ^18^, ^19^, ^20^, ^21^, ^22^, ^23^ macroscopic and significant (*P* < 0.05) improvements of OAR sparing and hot spot control were observed after substituting the objectives from patient‐specific estimations for the trial‐and‐error manual adjustment. The objective‐induced advantages were largely transferable from PO engine to the conventional PRO algorithm, yet the performance of PRO is slightly inferior than the PO. The more adjacent DVH lines between the PO (dotted) and PRO (dashed) than the clinical (solid) plans as shown in Fig. 1 echoed the aforementioned numerical observations.

Although RapidPlan is a purchasable package, PO algorithm should be available for all Eclipse users of version 13.5 or later. Therefore, it is of patients’ maximal benefit to select PO as the preferential optimizer for VMAT planning, even if knowledge‐based solution is not purchased or the models are not yet ready (custom model configuration is treatment site‐specific and sorely time‐consuming). The slight improvement of OAR sparing by a few percent may not be clinically significant, but it complies with “as low as reasonably achievable” (ALARA) principle at little or no extra cost of effort.

The lesser impact of the optimizer suggested that for the Eclipse users of older versions (before 13.5), transplanting the model‐estimated and patient‐specific objectives from other RapidPlan‐equipped centers can be a transitional option to improve the VMAT plan quality immediately even with PRO algorithm. Surely this should be performed with caution on the basis of consistent contouring/planning protocols, and thorough clinical validations.

The optimizers had little if any impact on the consumed MUs (Δ_PRO−PO_ = 1 MU, *P* = 0.81). However, MUs increased considerably and significantly (Δ > 32 MUs, *P* < 0.01) after the model‐estimated objectives were introduced, indicating higher modulation in PO and PRO plans than the clinical plans. More MUs might be associated with smaller MLC apertures and lower agreement between the calculation and delivery of the intended dose distribution.^24^, ^29^ Computation‐based analysis has excluded the inferior deliverability of knowledge‐based plans,^14^ yet physical measurement should still be desirable to gain more confidence. In addition, when more DVH estimation models become available, the comparative assessment could be performed for DVO algorithm and for other lesion sites.

This study is potentially limited by the nondeterministic nature of the optimization process: slightly different results may be obtained even if the same algorithm and constraints were utilized. However, this random and minor uncertainty should be largely canceled out by the averaging during the statistical analysis, and the systematic discrepancies should be mostly attributable to the different optimizers.

## 5 Conclusions

By differentiating the dosimetric improvement contributed by the knowledge‐based objectives and the new optimizer, this work suggests that PO prevails over PRO algorithm for VMAT planning, with or without RapidPlan solution.
